# An efficient interpolation technique for jump proposals in reversible-jump Markov chain Monte Carlo calculations

**DOI:** 10.1098/rsos.150030

**Published:** 2015-06-24

**Authors:** W. M. Farr, I. Mandel, D. Stevens

**Affiliations:** 1 Center for Interdisciplinary Exploration and Research in Astrophysics (CIERA), Department of Physics and Astronomy, Northwestern University, Evanston, IL, USA; 2 MIT Kavli Institute, Cambridge, MA, USA; 3 School of Physics and Astronomy, University of Birmingham, Birmingham, UK; 4 Monash Center for Astrophysics and School of Physics and Astronomy, Monash University, Clayton, Victoria, Australia; 5 Department of Astronomy, The Ohio State University, Columbus, OH, USA

**Keywords:** Markov chain Monte Carlo, reversible-jump Markov chain Monte Carlo, data analysis

## Abstract

Selection among alternative theoretical models given an observed dataset is an important challenge in many areas of physics and astronomy. Reversible-jump Markov chain Monte Carlo (RJMCMC) is an extremely powerful technique for performing Bayesian model selection, but it suffers from a fundamental difficulty and it requires jumps between model parameter spaces, but cannot efficiently explore both parameter spaces at once. Thus, a naive jump between parameter spaces is unlikely to be accepted in the Markov chain Monte Carlo (MCMC) algorithm and convergence is correspondingly slow. Here, we demonstrate an interpolation technique that uses samples from single-model MCMCs to propose intermodel jumps from an approximation to the single-model posterior of the target parameter space. The interpolation technique, based on a kD-tree data structure, is adaptive and efficient in modest dimensionality. We show that our technique leads to improved convergence over naive jumps in an RJMCMC, and compare it to other proposals in the literature to improve the convergence of RJMCMCs. We also demonstrate the use of the same interpolation technique as a way to construct efficient ‘global’ proposal distributions for single-model MCMCs without prior knowledge of the structure of the posterior distribution, and discuss improvements that permit the method to be used in higher dimensional spaces efficiently.

## 1. Introduction

Selection among alternative theoretical models given an observed dataset is an important challenge in many areas of physics and astronomy. In a Bayesian context, model selection involves computing the evidence for the data given each model. The model evidence is an integral of the unnormalized posterior probability distribution over the model parameter space, representing the probability of obtaining the dataset within that model. Models with larger evidence are preferred; the ratio of the evidences of two models is the Bayes factor between them. The product of the Bayes factor and the ratio of prior probabilities for the two models yields the odds ratio for the models.

There are many ways to compute model evidences. In low-dimensional parameter spaces, the unnormalized posterior probability can be evaluated on a grid or lattice and the integral can be performed directly. For many problems or models of interest, however, the dimensionality of the parameter space is too large to make this approach practical, and stochastic sampling must be used.

Markov chain Monte Carlo (MCMC) methods attempt to stochastically produce parameter samples with density proportional to the posterior probability distribution. In MCMC techniques, the primary target is an accurate estimate of the posterior distribution. (We note that an alternative stochastic method for exploring a model parameter space, nested sampling [1–3], focuses on evidence computation rather than sampling the posterior probability density functions.) It is not straightforward to compute the model evidence from MCMC samples. The most direct way to estimate the evidence for a model from MCMC samples is to compute the harmonic-mean estimator, but this estimator of the evidence can suffer from infinite variance [4–7]. MCMC implementations with parallel tempering [8,9] allow for evidence computation via thermodynamic integration [10], but these can be computationally costly.

Weinberg [11] gives a method for directly computing the evidence integral from existing MCMC samples by using a kD-tree data structure to decompose a parameter space into boxes containing the MCMC sample points. The integral is approximated as a sum over box volumes. This method is promising, but it is not clear in general what statistical and systematic errors it introduces and how these are affected by the shape of the posterior distribution which the MCMC samples.

When the goal is model selection between several known models, only the *relative* evidence of each model is needed. In this circumstance, the reversible-jump MCMC (RJMCMC) technique first introduced in [12] is one of the most reliable and accurate ways to compare the models. RJMCMC, described more fully in §2, performs a standard MCMC in space that is an augmented union of all the model parameter spaces. Such an MCMC involves both intra- and intermodel jumps; the number of MCMC samples in each model's parameter space is proportional to that model's relative evidence in the suite of models being compared.

Implemented naively, RJMCMC has a significant drawback: because the chain of samples must be Markovian, only the current sample is available to the algorithm as it is choosing the next sample. Each time an RJMCMC transitions between models, the information about the choices of parameter values in the previous model is lost; subsequent jumps into that model must ‘start fresh’, and are correspondingly unlikely to be accepted, delaying convergence of the RJMCMC sample chain (see §2.3 for a caveat). Littenberg & Cornish [13] addressed this issue by proposing a new method for producing intermodel jumps in an RJMCMC that relies on interpolating single-model posterior distributions using a box decomposition of parameter space.

Here, we introduce an alternative technique based on a kD-tree data structure to construct an approximation to each model's posterior parameter distribution. This improved interpolation method leads to faster convergence of RJMCMC sample chains. We draw jump proposals into the model from this approximation to its posterior. Because jumps are proposed preferentially to locations favoured by the single-model posterior, the RJMCMC compares ‘good’ locations in parameter space across all the models, and convergence is generally rapid. We have successfully applied this RJMCMC technique to a 10-way model selection among alternative mass distribution models for black-hole X-ray binaries [14]. We also provide an example using this method as an ‘asymptotically Markovian’ [15] jump proposal in the context of a single-model, nine-dimensional MCMC in §5.

The method of Littenberg & Cornish [13] for producing intermodel jumps in an RJMCMC relies on a box decomposition of parameter space, using fixed-sized boxes. The method cannot adapt to the local structure of the posterior, and becomes asymptotically inefficient for high-dimensional parameter spaces or highly peaked posteriors. Meanwhile, the approximation to the posterior distribution produced by the kD-tree is a constant-in-box interpolation of the posterior, similar in spirit to the phase-space density interpolants produced from N-body positions and momenta in [16]. The kD-tree interpolation is effective in parameter spaces of modest dimensionality and is quite space-efficient, requiring O(N) storage space and O(log⁡N) time to produce each proposed jump, where *N* is the number of samples in an MCMC over the parameter space of one model (‘single-model MCMC’) used to construct the interpolation.

The structure of this paper is as follows. In §2, we introduce in more detail the concept of an RJMCMC, and describe the fundamental difficulty with a naive jump proposal in an RJMCMC. In §3, we introduce the kD-tree data structure used to decompose the parameter space into boxes for interpolation. In §4, we demonstrate the efficiency gains that are achieved from use of the interpolated jump proposal. In §5, we give examples of some other uses of the interpolated jump proposal that suggest its utility in the context of a single-model MCMC. Finally, in §6, we offer a summary and some concluding remarks on the method.

## 2. Reversible-jump Markov chain Monte Carlo

RJMCMC [12] is a technique for Bayesian model comparison. Below, we give a very brief introduction to Bayesian analysis, describe a standard MCMC and introduce RJMCMC.

### 2.1 Bayesian analysis

Consider an observed dataset *d* and a set of competing models for the data, indexed by an integer *i*: {*M*_*i*_|*i*=1,2,…}. Each model has some continuous parameters, ***θ***_*i*_; given the model and its parameters, we can make a prediction about the likelihood of observing the experimental data: *L*(*d*|***θ***_*i*_,*M*_*i*_). Within the framework of each model, Bayes' rule gives us a way to compute the posterior probability distribution function (PDF) for the model parameters implied by the data:

2.1 $$p(\theta_{i}|d,M_{i})=\frac{L(d|\theta_{i},M_{i})p(\theta_{i}|M_{i})}{p(d|M_{i})},$$

where *p*(***θ***_*i*_|*d*,*M*_*i*_) is the posterior distribution for the model parameters ***θ***_*i*_ implied by the data in the context of model *M*_*i*_, *p*(***θ***_*i*_|*M*_*i*_) is the prior probability of the model parameters that represents our beliefs before accumulating any of the data *d*, and *p*(*d*|*M*_*i*_), called the evidence, is an overall normalizing constant which ensures that *p*(***θ***_*i*_|*d*,*M*_*i*_) is properly normalized as a probability distribution on the ***θ***_*i*_. This implies that the evidence is equal to

2.2 $$p(d|M_{i})=\int_{V_{i}}d\theta_{i}L(d|\theta_{i},M_{i})p(\theta_{i}|M_{i}),$$

where *V*_*i*_ is the parameter space volume in model *M*_*i*_. For model comparison, we are interested in the posterior probability of a particular model, *M*_*i*_, given the data, *p*(*M*_*i*_|*d*). Using Bayes' rule, we see that this involves the evidence, equation (2.2):

2.3 $$p(M_{i}|d)=\frac{p(d|M_{i})p(M_{i})}{p(d)},$$

where *p*(*M*_*i*_) is our *a priori* belief in model *M*_*i*_ and *p*(*d*) is a normalizing constant

2.4 $$p(d)=\sum_{i}p(d|M_{i})p(M_{i}).$$

When selecting among alternative models, we are interested in finding the model with the highest posterior probability *p*(*M*_*i*_|*d*). However, attempts to directly compute the evidence by performing the integration in equation (2.2) are generally very difficult in a multi-dimensional, multi-modal parameter space when the likelihood has to be evaluated numerically. In particular, a grid-based integral quickly becomes computationally unfeasible as the dimensionality of ***θ*** exceeds a few. The parameter space must typically be explored in a stochastic manner before the evidence integral can be computed. There are several stochastic parameter-exploration techniques focused directly on evidence computation (e.g. nested sampling [1,2] and its variant MultiNest [3]). Although nested sampling can be used to compute the posterior PDFs within each model along with the evidences for the various models, the most common technique for computing posterior PDFs in the context of a model is the Markov chain Monte Carlo, which we now describe.

### 2.2 Markov chain Monte Carlo

A Markov chain Monte Carlo [17] produces a set of samples {***θ***^(*j*)^ | *j*=1,…} from the model parameter space that are sampled according to the posterior, meaning that, in the limit that the chain length tends to infinity, the relative frequency with which a given set of parameters appears in the chain is proportional to the desired posterior, *p*(***θ***|*d*,*M*). Therefore, the output of an MCMC can be directly interpreted as the posterior PDF over the full parameter space, while PDFs for individual parameters can be obtained by marginalizing over the uninteresting parameters.

A Markov chain has the property that the probability distribution of the next state can depend only on the current state, not on the past history:

2.5 $$p(\theta^{\left(j+1\right)})=\int_{V}d\theta^{\left(j\right)}p(\theta^{\left(j\right)}\to \theta^{\left(j+1\right)})p(\theta^{\left(j\right)}),$$

where the jump probability *p*(***θ***^(*j*)^→***θ***^(*j*+1)^) depends only on ***θ***^(*j*)^ and ***θ***^(*j*+1)^. An additional requirement for an MCMC arises from the fact that the desired distribution is the equilibrium distribution. Detailed balance requires that *p*(***θ***^(*i*)^)*p*(***θ***^(*i*)^→***θ***^(*j*)^)=*p*(***θ***^(*j*)^)*p*(***θ***^(*j*)^→***θ***^(*i*)^).

One way to produce such a sequence of samples is via the Metropolis–Hastings algorithm, first proposed in [18], and later generalized in [19]:

(i) given a current state ***θ***^(*j*)^, propose the next state ***θ***^*p*^ by drawing from a jump proposal distribution with probability *Q*(***θ***^(*j*)^→***θ***^*p*^);
(ii) compute the probability of accepting the proposed jump as
  2.6 $$p_{\mathrm{accept}}\equiv \min\left(1,\frac{p(\theta^{p}|d,M)}{p(\theta^{\left(j\right)}|d,M)}\frac{Q(\theta^{p}\to \theta^{\left(j\right)})}{Q(\theta^{\left(j\right)}\to \theta^{p})}\right);$$
(iii) pick a uniform random number *α*∈[0,1]. If *α*<*p*_accept_, accept the proposed jump, setting ***θ***^(*j*+1)^=***θ***^*p*^. Otherwise, reject the jump, and remain at the same location in parameter space for the next step, ***θ***^(*j*+1)^=***θ***^(*j*)^.

This jump proposal distribution *Q*(***θ***^(*j*)^→***θ***^*p*^) can depend on the parameters of the current state ***θ***^(*j*)^, but not on the past history. It must also allow any state within the prior volume to be reachable (eventually) by the MCMC. Any jump proposal that satisfies these properties is suitable for an MCMC.

The jump proposal is the most important choice in the MCMC, as it determines the sampling efficiency of the algorithm, i.e. the length of the chain before it converges to the posterior PDF. Creating an efficient jump proposal distribution requires an understanding of the structure of the parameter space which may not be available until the PDFs are found, creating a catch-22; one possibility for resolving this infinite loop is described in §5.

It should be noted that although an MCMC whose jump acceptance criterium obeys detailed balance (as the Metropolis–Hastings algorithm does) must eventually converge to the desired distribution, there is no way to guarantee convergence in a fixed number of steps or to test whether a chain has converged in a foolproof manner. For example, MCMC chains can get stuck on local maxima, producing an apparently well-converged sampling of the PDF in the vicinity of the maximum; or, if the chain visits a sequence of local maxima, moving rarely between maxima, the autocorrelation length of the chain may represent a substantial fraction of the total number of samples, resulting in an effective sample size that is too small to accurately represent the relative sizes of the modes in the PDF.

Finally, we note that, in practice, the randomly chosen initial starting point of the MCMC may be in a particularly unlikely location in the parameter space. Because jumps are frequently local, we will generally want to ignore the early samples in a finite-size chain to avoid biases in the recovered posterior PDF owing to the choice of the initial location. The samples thus discarded are referred to as ‘burn-in’ samples.

### 2.3 Reversible-jump Markov chain Monte Carlo

The samples produced by an MCMC algorithm can be used to directly perform a Monte Carlo evidence integral. This results in a harmonic mean estimator for the evidence, which may suffer from infinite variance [4–7]. Additional techniques for the direct integration of evidence, also based on a kD tree decomposition of the parameter space (see §3), are described in [11]. These techniques are promising, but in some cases suffer from large variance and bias [14]. An alternative approach to model selection among a set of models is based on performing an MCMC in a ‘super-model’ that encompasses all of the models under consideration; this is known the RJMCMC.

The parameter space of the super-model in an RJMCMC consists of a discrete parameter that identifies the model, *M*_*i*_, and a set of continuous parameters appropriate for that model, ***θ***_*i*_. Thus, each sample consists of a model identifier and a location within the parameter space of that model, {*M*_*i*_,***θ***_*i*_}. We perform the MCMC in the ‘super-model’ parameter space just like a regular MCMC; we propose jumps to different parameters within a model (intramodel jumps) and jumps to a different model with different parameters (intermodel jumps). The acceptance probability for a proposed jump from $\theta_{i}^{\left(j\right)}$ in model *M*_*i*_ to $\theta_{j}^{p}$ in model *M*_*j*_ becomes

2.7 $$p_{\mathrm{accept}}\equiv \min\left(1,\frac{p(\theta_{j}^{p},M_{j}|d)}{p(\theta_{i}^{\left(j\right)},M_{i}|d)}\frac{Q(\theta_{j}^{p},M_{j}\to \theta_{i}^{\left(j\right)},M_{i})}{Q(\theta_{i}^{\left(j\right)},M_{i}\to \theta_{j}^{p},M_{j})}\right).$$

Here, the *Q* factors incorporate both a discrete probability on the model index, reflecting the probabilistic choice of which model to jump into, and also a continuous probability density on target model's parameter space. For example, $Q(\theta_{j}^{p},M_{j}\to \theta_{i}^{\left(j\right)},M_{i})$ has a factor for the probability of proposing a jump to model *i* when in model *j* and is a density on the parameter space of *M*_*i*_. These densities cancel the corresponding densities in the ratio of posteriors, making the acceptance probability a parametrization-independent scalar. In the common special case where the two parameter spaces have equal dimension and the jump proposal is a diffeomorphism, *ϕ*, between them,

2.8 $$\theta_{j}^{p}=\phi (\theta_{i}^{\left(j\right)}),$$

and therefore

2.9 $$\theta_{i}^{\left(j\right)}=\phi^{-1}(\theta_{j}^{p}),$$

then the jump proposal ratio reduces to the Jacobian of the diffeomorphism:

2.10 $$\frac{Q(\theta_{j}^{p},M_{j}\to \theta_{i}^{\left(j\right)},M_{i})}{Q(\theta_{i}^{\left(j\right)},M_{i}\to \theta_{j}^{p},M_{j})}=\left|\frac{\partial \phi }{\partial \theta_{i}^{\left(j\right)}}\right|.$$

The resulting chain samples from the posterior *p*(*M*_*i*_,{***θ***_*i*_}|*d*). As in a usual MCMC, the PDF on the model as a parameter, with other parameters ignored, is obtained by marginalizing over the remaining parameters. The posterior probability of a model is proportional to the number of counts:

2.11 $$p(M_{i}|d)=\int d\theta_{i}\frac{L(d|M_{i},\theta_{i})p(\theta_{i}|M_{i})p(M_{i})}{p(d)}\approx \frac{N_{i}}{N},$$

where *N*_*i*_ is the number of RJMCMC samples listing the *i*'th model and *N* is the total chain length. Thus, the probability of a particular model relative to other models under consideration is given by the fraction of RJMCMC samples lying in the parameter space of that model.

The main difficulty of achieving an efficient RJMCMC is finding a good jump proposal distribution for intermodel jumps. In order to have relatively high acceptance ratios for intermodel jumps, which is necessary for efficient mixing between models, jumps should be preferentially proposed into regions with a high posterior. However, because the algorithm is Markovian, it has no past memory, so a jump proposed into a model from outside cannot access information from earlier in the chain which may identify a posterior peak. It is, in principle, possible to overcome this constraint by storing a union of {***θ***_*i*_} as the MCMC state vector, with the likelihood a function only of parameters ***θ***_*i*_ that correspond to the current model *M*_*i*_. In this case, intermodel jump proposals would change only the model *M*_*i*_. However, if the chain finds a high-likelihood region in one model space faster than in another, a jump to the other model will take a very long time to be accepted—again rendering RJMCMC inefficient.

The way to solve this problem is to identify a good jump proposal distribution in advance, by exploiting information from single-model MCMCs to generate efficient jump proposal distributions for our RJMCMC (single-model MCMCs can take small local jumps within their model, meaning that they are much less likely than an RJMCMC to lose a high-posterior mode once it has been located). The ideal jump proposal distribution for the parameters within a model would consist of the posterior PDF for those parameters, *p*(***θ***_*i*_|*M*_*i*_,*d*), and single-model MCMCs already represent samples from these posterior PDFs. However, the samples are discrete, and a jump proposal must be continuous. Therefore, the output of each single-model MCMC must be interpolated to construct the desired jump proposal. The strategy we propose for efficiently interpolating a discretely sampled PDF is described in the next section.

## 3. kD-trees and interpolation

The problem of drawing a proposed jump from an interpolation of single-model MCMC samples can be thought of as the problem of assigning a local ‘neighbourhood’ to each sample in the MCMC chain. We choose these neighbourhoods to be non-overlapping and to fill the parameter space. The size of a neighbourhood is inversely proportional to the local sample density. The proposed jumps are drawn from a piecewise-constant (constant on each neighbourhood) interpolation of the PDF. To draw a proposed jump, we select a sample uniformly from the MCMC samples, find its associated neighbourhood, and then draw the proposed jump uniformly from the neighbourhood. Since the MCMC samples are distributed according to the posterior PDF for the single model, this procedure produces proposed jumps that are approximately distributed according to the posterior PDF.

There are various techniques that could be used to construct the set of neighbourhoods associated with each sample. Littenberg & Cornish [13] decompose the parameter space into constant-volume ‘bricks’ whose size is set by the typical size of the peaks of the PDF. Each sample is associated with the brick that contains it, and the probability of proposing a jump into a particular brick is thus proportional to the number of samples within that brick. Additionally, an extra uniform jump proposal is added to allow for jumps into bricks that do not contain any samples, so that the jump proposal covers the entire model parameter space. However, the bricks in this algorithm do not adapt to the local structure of the PDF. One must either use small bricks to capture the local structure of the PDF, placing many bricks in regions without MCMC samples (which can increase memory management and access costs), or use large bricks, missing the local structure of the PDF in exchange for fewer empty bricks.

An alternate technique for producing adaptive neighbourhoods would be to use the Voronoi regions [20] associated with each MCMC sample. The Voronoi region associated with a sample contains all the parameter space points that are closer to that sample than any other sample. The Voronoi region decomposition into neighbourhoods is, in a sense, maximally adaptive, in contrast to the approach of [13], which is minimally adaptive. Unfortunately, defining the Voronoi decomposition requires a metric on parameter space, which may be difficult or impossible to define. In addition, the computational cost for computing the Voronoi regions increases rapidly with dimensionality.

Here, we propose to use a decomposition of the parameter space into neighbourhoods based on a data structure called a kD-tree (e.g. [21] or [22]). The decomposition is more adaptive than the boxes of [13], and more efficient in high-dimensional spaces than the Voronoi decomposition.

A kD-tree is a binary, space-partitioning tree. To partition a set of samples into a kD-tree, begin by placing them in a rectangular box that contains all of parameter space. Then proceed recursively^1^ :

(i) if the given box contains exactly one sample, stop; this is a leaf of the tree. Otherwise:
(ii) choose a dimension along which to divide the samples. Divide the samples in half along this dimension (or nearly in half, if the number of samples is odd), forming two sub-boxes. The ‘left’ sub-box contains the half (or nearly half) of the samples that have small coordinates along the chosen dimension; the ‘right’ sub-box contains the half (or nearly half) of the samples that have large coordinates along the chosen dimension;
(iii) return to step (i) with each of the sub-boxes, storing the resulting trees as sub-trees of the current box.

**Figure 1.**
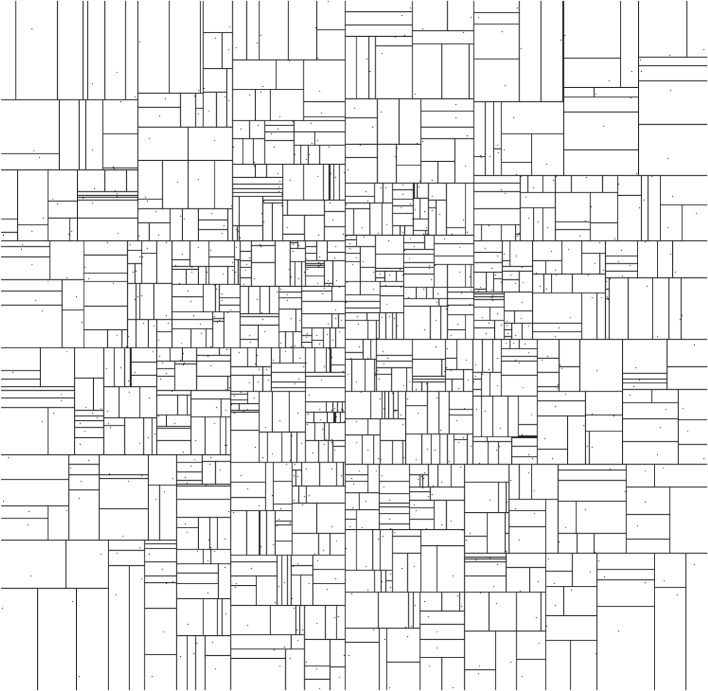
The neighbourhoods from a kD-tree constructed around a set of samples that are normally distributed about the origin in two dimensions. As the samples become denser around the origin, the typical neighbourhood gets smaller. The interpolated PDF within a box of volume *V*_*i*_ is 1/(*NV*_*i*_), where *N* is the total number of samples (which is also the number of boxes).

The key algorithmic step in the production of a kD-tree is finding the median sample along a given dimension in order to divide the samples in half in step (ii). For *n* samples, this can be accomplished in O(n) time (e.g. [23]). If there are *N* samples in total, there are O(log⁡N) levels in the tree; at each level, O(N) samples must be processed once in the median-finding algorithm. Tree construction thus costs O(Nlog⁡N) in time, and the tree consumes O(N) space. As an illustration, box boundaries for a kD-tree constructed around a sample set that is normally distributed around the origin in two dimensions are shown in figure 1.

In order to use the kD-tree interpolation as a jump proposal in an MCMC, we randomly select a point stored in the kD tree with equal probability, and propose from the associated neighbourhood. Therefore, we must be able to quickly find the neighbourhood associated with a given point to compute the jump probability (see equation (2.6)). We introduce an additional parameter into the neighbourhood search, *N*_boxing_, which describes the minimum number of points in a kD box used as the neighbourhood for a point; small *N*_boxing_ increases variance in the proposal but resolves finer scale structure in the posterior. The neighbourhood search can be accomplished in O(log⁡N) time and constant space with the following algorithm, which is a modified binary tree search. Given the randomly chosen point, ***θ***_*i*_, the tree, *T* and *N*_boxing_:

(i) if *T* contains fewer than 2*N*_boxing_ points, then its box is the associated neighbourhood. Otherwise:
(ii) the tree *T* has two sub-trees. If the point ***θ***_*i*_ is contained in the ‘left’ sub-tree, then return to step (i), considering this sub-tree; otherwise return to step (i), considering the ‘right’ sub-tree.

The returned box is used for the jump proposal by drawing uniformly from its interior, so the proposal density is

3.1 $$Q(\theta \to \theta^{p})=\frac{N_{\mathrm{box}}}{NV},$$

where *N* is the number of points in the tree, *N*_box_ is the number of points in the chosen kD box and *V* is the coordinate-space volume of the box.

## 4. Reversible-jump Markov chain Monte Carlo efficiency

In this section, we demonstrate the efficiency of the kD-interpolated jump proposal on a toy model-comparison problem. The same algorithm has been used in real-world settings [14] and, as discussed below, is available in several forms as a software library for re-use by others.

In the toy model of this section, we draw *N*=100 simulated data points from a *N*(0,1) Gaussian distribution, and then ask whether these data are better described by a model where they are Gaussian distributed with unknown mean *μ* and standard deviation *σ*:

4.1 $$p(x)=\frac{1}{\sqrt{2\pi }\sigma }\exp\left(-\frac{{\left(x-\mu \right)}^{2}}{2\sigma^{2}}\right),$$

or by a model where they are Cauchy distributed with mode *α* and width *β*:

4.2 $$p(x)=\frac{1}{\pi \beta \left(1+{\left((x-\alpha )/\beta \right)}^{2}\right)}.$$

We take priors on *μ* and *α* to be uniform in [−1,1], and priors in *σ* and *β* to be uniform in [0.5,1.5]. With a dataset of 100 points, the relative uncertainty in determining the parameters of the underlying distribution is approximately 10%, so we expect the posterior probabilities in the (*μ*,*σ*) and (*α*,*β*) spaces to occupy only a few per cent of the prior volume. The Cauchy distribution is much broader than the Gaussian (it has no finite moments), so with equal model priors, the posterior probability for the Gaussian model over the Cauchy model is extremely large:

4.3 $$\frac{p(\text{Gaussian}|d)}{p(\text{Cauchy}|d)}∼10^{9}.$$

In order to ensure that the RJMCMC produces samples in the Cauchy model at all, we impose a model prior that favours the Cauchy model by 5×10^8^ relative to the Gaussian. The evidence ratio between the models for our chosen dataset with these priors is

4.4 $$\frac{p(\text{Gaussian}|d)}{p(\text{Cauchy}|d)}\equiv r=1.15,$$

yielding a theoretical maximum acceptance rate of intermodel jumps of (1+1/*r*)/2=0.93.

**Figure 2.**
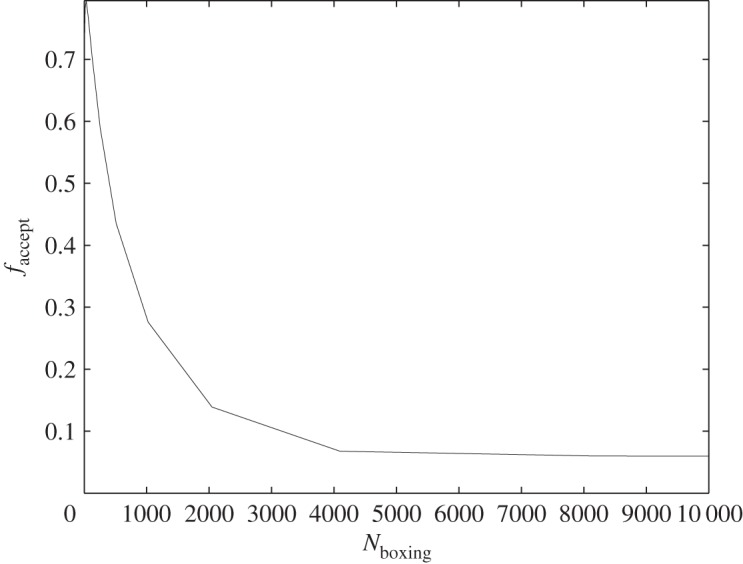
The intermodel jump acceptance rate versus the number of samples per box when the kD-tree neighbourhood search is truncated. As the number of samples per box increases, and the interpolation becomes less accurate, the acceptance rate falls, asymptoting to the rate for naive draws from the uniform prior (about 5% for this dataset).

We obtain 10^4^ single-model MCMC samples by independently running MCMC within each model, and use the kD-tree interpolation method described above to propose intermodel jumps in an RJMCMC. The acceptance rate of intermodel jumps is approximately 0.8. To explore how the efficiency of the method degrades as the interpolation becomes less accurate, we artificially truncated the kD-tree with higher and higher numbers of samples in each box (this can be accomplished during the neighbourhood search phase by stopping the search for a box when one is found containing the desired number of samples). For each truncation choice, we performed an RJMCMC with the resulting interpolated jump proposal. The acceptance rate is plotted against the number of single-model MCMC samples per box (kD-tree leaf) in figure 2. The more samples in each leaf of the tree when the search is truncated, the lower the acceptance probability; when points are drawn from the top level of the tree, the acceptance probability asymptotes to the naive draw from the prior (approx. 5%).

The relative error on the determination of the Bayes factor (evidence ratio) scales with $1/\sqrt{N_{\mathrm{transitions}}}$, where *N*_transitions_ is the number of intermodel transitions in the RJMCMC. Thus, as the acceptance rate of intermodel jumps goes down, the RJMCMC must run longer to achieve a desired accuracy in the evidence ratio. By boosting the acceptance rate of intermodel jumps, the interpolation method described above can improve the runtime of an RJMCMC.

## 5. kD-interpolated jump proposal in higher dimensional single-model Markov chain Monte Carlo

In the model selection example from §4, the models have two-dimensional parameter spaces; in [14], the highest dimensional model had five dimensions. As the number of dimensions increases, the interpolation becomes more difficult for two reasons. First, the number of samples required for a given number of subdivisions in each dimension grows exponentially with the number of dimensions; hence, for a reasonable number of samples, a high-dimensional kD-tree will have few subdivisions along each dimension. Second, as the dimensionality increases, the fraction of the volume at the ‘edges’ and ‘corners’ of each kD-tree box becomes more significant, and the placement of the sample becomes increasingly unrepresentative of the typical density in the box. This problem is even more pronounced when the distribution of samples does not align with the coordinate axes along which the tree subdivides.

In this section, we propose a practical solution that allows us to incorporate larger scale features in the sample density distribution. We illustrate our solution with an single-model MCMC that updates the jump proposal distribution on-the-fly by using a kD-tree to interpolate the density of existing samples. We conclude with an example of a successful application to gravitational-wave parameter estimation.

**Figure 3.**
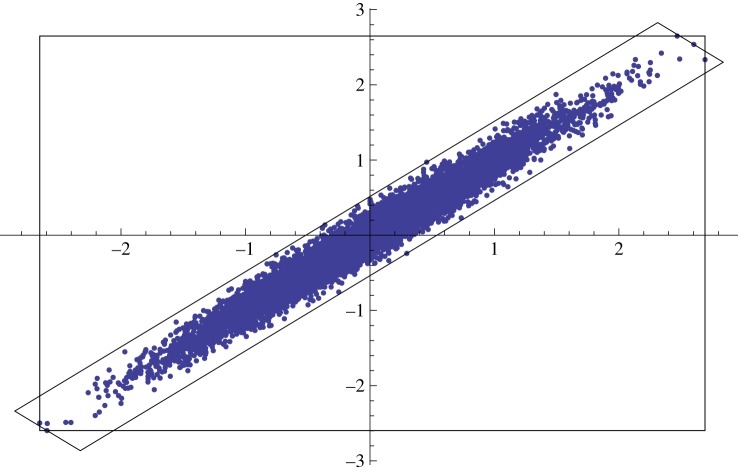
A two-dimensional illustration of the two boxes involved in the modified kD proposal. The larger box aligned with the axes is the normal kD-tree box containing the given samples, while the tighter box is the ‘covariance cell’ aligned with the eigenvectors of the sample distribution. The modified kD-interpolated jump proposal draws a point from the intersection of the two boxes. Tightly correlated posteriors in parameter space such as this are typical of the gravitational-wave parameter estimation problem described in [24] and in the text. Without the modification to account for the correlated samples, the kD neighbourhood of these points would produce a very inefficient jump proposal, as most of the bounding-box would contain empty (i.e. low-posterior) parameter space.

The kD-tree-based interpolated jump proposal described in §3 selects one sample from the tree at random and proposes a uniform point from the box containing that sample. This ignores the density of other samples in the neighbourhood of this box, which contains additional information about the true density within the box, which is probably non-uniform. A better proposal, therefore, would account for larger scale sample covariances in parameter space. To address significant covariance between samples, which would correspond to strong density gradients and a very non-uniform density profile within a rectangular box, the modified jump proposal does not descend to the leaf-nodes of the tree, but instead stops descending whenever the number of samples in the current box, *N*_box_, falls below some threshold, *N*_crit_. We then find the principal axes of the samples contained in this box by calculating the eigenvectors of the covariance matrix of these samples. We use these eigenvectors to draw a new box. This box—henceforth called a covariance cell—contains all *N*_box_ samples from the kD-tree box, is centred on the mean of these samples and has edges that are aligned with the principal axes. Furthermore, this box is drawn around the samples as tightly as possible; in other words, for each edge, there exists a point in the covariance cell that lies along that edge. Figure 3 illustrates these two boxes.

The jump proposal chooses a stored sample at random and finds the largest kD-tree box containing it and fewer than *N*_crit_ total samples. It then draws the covariance cell and proposes a new point uniformly from the intersection of the covariance cell with the original kD-tree box. The jump probability for this jump proposal is still (cf. equation (3.1)) given by

5.1 $$Q(\theta_{i}\to \theta_{i+1})=\frac{N_{\mathrm{box}}}{NV},$$

where *N* is the total number of samples in the kD-tree, and *V* is the volume of the intersection between the kD-tree box and the correlation cell containing ***θ***_*i*+1_. When used as the only jump proposal for an RJMCMC, it is important that this proposal be capable of proposing points in all allowed regions of parameter space; on the other hand, when the kD proposal is used in a suite of other jump proposals, this is no longer a requirement.

This technique trivially violates detailed balance since the jump proposal distribution depends on the past history. One way to address this problem, through diminishing adaptations [25], requires continuously accumulating samples into the kD-tree as the MCMC explores more of the parameter space. As the number of samples drawn from the posterior PDF increases, the neighbourhoods around each sample decrease with increasing sample density and the calculated covariances between samples in the kD-tree better approximate the true covariances between parameters in the equilibrium posterior PDF. Hence, for large *N*, the change in $Q(\theta^{\left(j\right)}\to \theta^{\left(j+1\right)})$ approaches zero and the jump proposal does a progressively better job of sampling the equilibrium posterior while becoming asymptotically Markovian. Conservatively, the entire set of samples obtained while the jump proposal is being dynamically updated could be discarded as burn-in, and future analysis could use the static jump proposal distribution interpolated from samples accumulated during the extended burn-in phase.

To efficiently insert samples into the tree as the chain accumulates them, the algorithm from §3 must be modified:

(i) instead of partitioning the sample set at its median, we now partition the bounding box at its geometric centre along a particular dimension, which cycles as we descend the tree levels. Note that this allows for empty boxes if all the samples cluster to one side of the domain; and
(ii) when inserting a sample, we descend the tree to a leaf node, which now can be either empty or contain one sample. If it is empty, we insert the sample at this node. If it contains one sample, we subdivide the appropriate dimension, and place both samples into the two sub-boxes. If both land in the same sub-box, we continue subdividing, until each box in the sub-sub-sub… tree contains one or zero samples.

We have implemented this modified kD-tree proposal as one of many jump proposals in the LALInferenceMCMC sampler [24,26–28]. LALInferenceMCMC is a MCMC code, based on the LIGO algorithms library (http://www.lsc-group.phys.uwm.edu/lal), designed to sample the posterior on parameters of merging compact-object binaries (masses, sky location, orbital orientation, distance, etc.) encoded in their gravitational-wave signatures as observed by the ground-based gravitational-wave detectors LIGO [29] and Virgo [30]. The simplest such signal has a nine-dimensional parameter space, and the posterior often includes near-degeneracies and multiple modes that make convergence of the MCMC chain very slow with traditional proposals [31].

**Figure 4.**
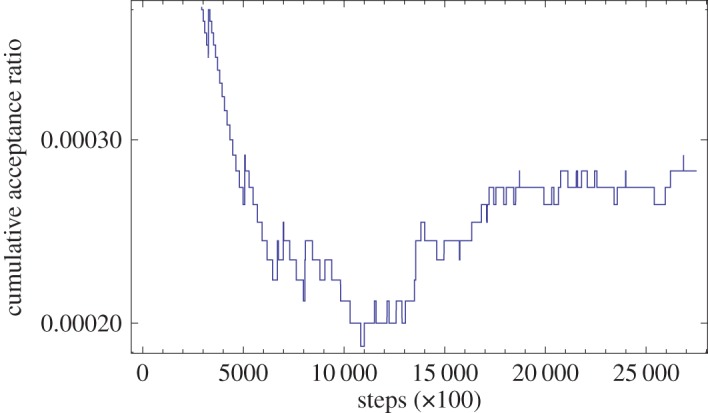
The cumulative acceptance ratio for the modified kD-tree jump proposal used in the LALInferenceMCMC code as a function of the number of steps (in hundreds). The simulation in question was a nine-dimensional analysis of a simulated gravitational-wave signal injected into synthetic data similar to that taken by the LIGO and Virgo detectors [29,30]. The parameter space includes the masses of the compact objects generating the gravitational-wave, their location on the sky, distance, orbital orientation, the time of signal arrival and the orbital phase. The posterior in this problem has a number of well-separated modes in the parameter space which are difficult to jump between using traditional jump proposals; in spite of the small acceptance ratio of the kD proposal, when applied to one in 20 jumps proposed in this simulation, it improved the convergence time of the sampler by a factor of two compared to using only the standard suite of proposals. The acceptance rate asymptotes to the steady-state solution once sufficient samples have been accumulated in the kD-tree to allow the sample density to be accurately interpolated; samples collected prior to this point should be discarded as burn-in.

Figure 4 shows the acceptance ratio for the kD-tree jump proposal applied as part of a LALInferenceMCMC analysis. In this example, the kD-tree jump proposal is one of several jump proposals used during the MCMC, and the kD-tree itself is updated with accepted samples whenever its jump proposal is called. In spite of the very low acceptance rate of the proposal, applying the kD-tree proposal to one out of 20 proposed jumps improved the convergence time—defined as the average time to reach a specified number of independent samples from the posterior, thinning by the autocorrelation length as described in [24]—of the simulation by a factor of two compared to the standard suite of proposals, because it is particularly efficient at producing ‘mode-hopping’ jumps, which are difficult to produce with the other proposals in LALInferenceMCMC.

## 6. Conclusion

The need to compare evidences for multiple models arises in a large variety of physical and astronomical contexts. In this paper, we described a technique that allows for efficient evidence computations via an RJMCMC. This technique solves the usual problem of finding good intermodel jump proposals in an RJMCMC by using a kD-tree to quickly and accurately interpolate an approximate posterior PDF from a single-model MCMC run, and then proposing efficient intermodel jumps from this interpolated PDF.

We demonstrated the efficiency of this technique on a toy model-comparison problem described in §4. We also successfully applied this technique to the problem of selecting the best model for the observed distribution of black-hole X-ray binary masses, as described in [14]. In addition to model comparison, the PDF interpolation described here can be useful in single-model MCMCs to inform the jump proposal distribution on-the-fly in order to propose jumps that can efficiently sample the parameter space (see §5), or to test MCMC convergence.

We have made our implementation of the technique described in this paper publicly available at http://github.com/farr/mcmc-ocaml, and also in the LALInferenceMCMC sampler, at https://www.lsc-group.phys.uwm.edu/daswg/projects/lalsuite.html. We welcome readers to take advantage of this toolkit.

## Footnotes

1 The kD-tree data structure defined here places box boundaries between the samples. An alternate definition common in the literature places box boundaries on the median sample, but such a definition is inconvenient for our purposes.

## References

[1] Skilling J 2004 Nested sampling (eds Fischer R, Preuss R, Toussaint UV). American Institute of Physics Conference Series, vol. 735, pp. 395–405.

[2] Skilling J 2006 Nested sampling for general Bayesian computation. Bayesian Anal. 1, 833–859. (doi:10.1214/06-BA127)

[3] Feroz F, Hobson MP, Bridges M 2009 MultiNest: an efficient and robust Bayesian inference tool for cosmology and particle physics. Mon. Not. R. Astron. Soc. 398, 1601–1614. (doi:10.1111/j.1365-2966.2009.14548.x)

[4] Newton MA, Raftery AE 1994 Approximate Bayesian inference with the weighted likelihood bootstrap. J. R. Stat. Soc. B 56, 3–48.

[5] Chib S 1995 Marginal likelihood from the Gibbs output. J. Am. Stat. Assoc. 90, 1313–1321. (doi:10.1080/01621459.1995.10476635)

[6] van Haasteren R 2009 Bayesian evidence: can we beat MultiNest using traditional MCMC methods? (http://arxiv.org/abs/0911.2150).

[7] Wolpert RL, Schmidler SC 2012 *α*-Stable limit laws for harmonic mean estimators of marginal likelihoods. Stat. Sinica 22, 1233–1251.

[8] Swendsen RH, Wang JS 1986 Replica Monte Carlo simulation of spin-glasses. Phys. Rev. Lett. 57, 2607–2609. (doi:10.1103/PhysRevLett.57.2607) 10033814 10.1103/PhysRevLett.57.2607

[9] Earl DJ, Deem MW 2005 Parallel tempering: theory, applications, and new perspectives. Phys. Chem. Chem. Phys. 7, 3910–3916. (doi:10.1039/b509983h) 19810318 10.1039/b509983h

[10] Friel N, Pettitt AN 2008 Marginal likelihood estimation via power posteriors. J. R. Stat. Soc. B 70,589–607. (doi:10.1111/j.1467-9868.2007.00650.x)

[11] Weinberg MD 2009 Computing the Bayesian factor from a Markov chain Monte Carlo simulation of the posterior distribution. (http://arxiv.org/abs/0911.1777).

[12] Green PJ 1995 Reversible jump Markov chain Monte Carlo computation and Bayesian model determination. Biometrika 82, 711–732. (doi:10.1093/biomet/82.4.711)

[13] Littenberg TB, Cornish NJ 2009 Bayesian approach to the detection problem in gravitational wave astronomy. Phys. Rev. D 80, 063007 (doi:10.1103/PhysRevD.80.063007)

[14] Farr WM, Sravan N, Cantrell A, Kreidberg L, Bailyn CD, Mandel I, Kalogera V 2011 The mass distribution of stellar-mass black holes. Astrophys. J. 741, 103 (doi:10.1088/0004-637X/741/2/103)

[15] ter Braak CJ, Frugt JA 2008 Differential evolution Markov Chain with snooker updater and fewer chains. Stat. Comput. 18, 435–446. (doi:10.1007/s11222-008-9104-9)

[16] Ascasibar Y, Binney J 2005 Numerical estimation of densities. Mon. Not. R. Astron. Soc. 356, 872–882. (doi:10.1111/j.1365-2966.2004.08480.x)

[17] Gilks WR, Richardson S, Spiegelhalter DJ 1996 Markov chain Monte Carlo in practice. London, UK: Chapman & Hall.

[18] Metropolis N, Rosenbluth AW, Rosenbluth MN, Teller AH, Teller E 1953 Equation of state calculations by fast computing machines. J. Chem. Phys. 21, 1087–1092. (doi:10.1063/1.1699114)

[19] Hastings WK 1970 Monte Carlo sampling methods using Markov chains and their applications. Biometrika 57, 97–109. (doi:10.1093/biomet/57.1.97)

[20] Voronoi G 1907 Nouvelles applications des paramètres continus à la thèorie des formes quadratiques. Journal für die Reine und Angewandte Mathematik 133, 97–178.

[21] de Berg M, Cheong O, van Kreveld M, Overmars M 2008 Computational geometry, 3rd edn Berlin, Germany: Springer.

[22] Gaede V, Günther O 1998 Multidimensional access methods. ACM Comput. Surv. 30, 170–231. (doi:10.1145/280277.280279)

[23] Press WH, Teukolsky SA, Vetterling WT, Flannery BP 2007 Numerical recipes: the art of scientific computing, 3rd edn Cambridge, UK: Cambridge University Press.

[24] Veitch J 2015 Parameter estimation for compact binaries with ground-based gravitational-wave observations using the LALInference software library. Phys. Rev. D 91, 042003 (doi:10.1103/PhysRevD.91.042003)

[25] Brooks S, Gelman A, Jones G, Meng X 2011 Handbook of Markov chain Monte Carlo. London, UK: Chapman and Hall.

[26] van der Sluys M, Raymond V, Mandel I, Röver C, Christensen N, Kalogera V, Meyer R, Vecchio A 2008 Parameter estimation of spinning binary inspirals using Markov chain Monte Carlo. Class. Quantum Gravity 25, 184011 (doi:10.1088/0264-9381/25/18/184011)

[27] Raymond V, van der Sluys MV, Mandel I, Kalogera V, Röver C, Christensen N 2010 The effects of LIGO detector noise on a 15-dimensional Markov-chain Monte Carlo analysis of gravitational-wave signals. Class. Quant. Grav. 27, 114009 (doi:10.1088/0264-9381/27/11/114009)

[28] Raymond V 2012 Parameter estimation using Markov Chain Monte Carlo methods for gravitational waves from spinning inspirals of compact objects. PhD thesis, Northwestern University, Evanston, IL, USA.

[29] Harry GM, the LIGO Scientific Collaboration. 2010 Advanced LIGO: the next generation of gravitational wave detectors. Class. Quant. Grav. 27, 084006 (doi:10.1088/0264-9381/27/8/084006)

[30] Virgo Collaboration. 2009 Advanced Virgo baseline design. Virgo Technical Report VIR-0027A-09. See https://tds.ego-gw.it/itf/tds/file.php?callFile=VIR-0027A-09.pdf.

[31] Aasi J 2013 Parameter estimation for compact binary coalescence signals with the first generation gravitational-wave detector network. Phys. Rev. D 88, 062001 (doi:10.1103/PhysRevD.88.062001)

